# Design Considerations for Massively Parallel Sequencing Studies of Complex Human Disease

**DOI:** 10.1371/journal.pone.0023221

**Published:** 2011-08-05

**Authors:** Bing-Jian Feng, Sean V. Tavtigian, Melissa C. Southey, David E. Goldgar

**Affiliations:** 1 Department of Dermatology, University of Utah School of Medicine, Salt Lake City, Utah, United States of America; 2 Huntsman Cancer Institute and Department of Oncological Sciences, University of Utah, Salt Lake City, Utah, United States of America; 3 Department of Pathology, University of Melbourne, Melbourne, Victoria, Australia; Aarhus University, Denmark

## Abstract

Massively Parallel Sequencing (MPS) allows sequencing of entire exomes and genomes to now be done at reasonable cost, and its utility for identifying genes responsible for rare Mendelian disorders has been demonstrated. However, for a complex disease, study designs need to accommodate substantial degrees of locus, allelic, and phenotypic heterogeneity, as well as complex relationships between genotype and phenotype. Such considerations include careful selection of samples for sequencing and a well-developed strategy for identifying the few “true” disease susceptibility genes from among the many irrelevant genes that will be found to harbor rare variants. To examine these issues we have performed simulation-based analyses in order to compare several strategies for MPS sequencing in complex disease. Factors examined include genetic architecture, sample size, number and relationship of individuals selected for sequencing, and a variety of filters based on variant type, multiple observations of genes and concordance of genetic variants within pedigrees. A two-stage design was assumed where genes from the MPS analysis of high-risk families are evaluated in a secondary screening phase of a larger set of probands with more modest family histories. Designs were evaluated using a cost function that assumes the cost of sequencing the whole exome is 400 times that of sequencing a single candidate gene. Results indicate that while requiring variants to be identified in multiple pedigrees and/or in multiple individuals in the same pedigree are effective strategies for reducing false positives, there is a danger of over-filtering so that most true susceptibility genes are missed. In most cases, sequencing more than two individuals per pedigree results in reduced power without any benefit in terms of reduced overall cost. Further, our results suggest that although no single strategy is optimal, simulations can provide important guidelines for study design.

## Introduction

Over the past two decades, advances in genome technology have greatly facilitated the discovery of genetic variation which confer increased susceptibility to disease, first through genetic maps of microsatellite markers which allowed mapping and then positional cloning of relatively high-penetrance disease predisposing mutations in genes such as *BRCA1* [MIM 113705] and *BRCA2* [MIM 600185], and then through the ability of high throughput, relatively low cost platforms containing 300,000–1,000,000 single nucleotide polymorphisms (SNPs) which have greatly facilitated the identification of common genetic variants conferring only modest increases in disease risk. To date, over 800 disease susceptibility loci in ∼150 human diseases/traits have been identified at genome-wide significance [1], thus validating this approach. In addition, less frequent, moderate-risk susceptibility alleles have been identified through resequencing of candidate genes selected on the basis of biological plausibility. However, it has also become clear that for many diseases and human traits, the known loci explain only relatively small fractions of the total genetic variance. While further genome-wide association studies using ever-higher density arrays, larger sample sizes and encompassing copy number variations will account for some of the missing genetic effect, it is unlikely that this proportion will increase markedly via this approach. The latest improvement in technology can be directed towards resolving this and identifying the missing heritability that seems a general phenomenon in many human diseases. There are several ways in which such technology can identify disease associated genetic variation; through resequencing of genomic regions implicated through GWAS in the hope/expectation of identifying rarer variants associated with higher risk that are tagged by the SNPs arrayed in the GWAS platforms [2], and through the possible identification of rare high-penetrance mutations in several disease susceptibility genes. Although it is impossible to predict which of the above hypotheses are correct and it is possible that the missing heritability will be explained by a combination of them all, it is clear that this may now be tested through the latest genomic technology. Massively Parallel Sequencing (MPS) provides order of magnitude improvement in throughput over Sanger sequencing enabling “genome-wide” sequencing applications in single sample preparations.

Recently, Ng et al. elegantly demonstrated the utility of whole-exome massively parallel sequencing in the identification of a gene for a rare Mendelian disorder (Miller Syndrome) based on analysis of only four unrelated individuals [3]. Here the authors benefited from the fact that not only did the four individuals have mutations in the same gene, but that all four had the same mutation. This considerably facilitated the task of proving that these mutations were in fact causal for the disorder. In another case it was the observation of multiple (causal) variants in the same gene that was the key observation leading to the identification of the susceptibility gene [4]. Subsequently whole-exome sequencing has been used to identify genes for other rare Mendelian diseases whose genetic basis was previously unknown (reviewed in Ng et al. [5]), typically using only a small number of individuals. However, to date, the success in rare Mendelian diseases has not been replicated in common diseases, even those with a demonstrated large genetic component.

How then can we best use these new sequencing capabilities to find rare high-penetrance disease-associated variants for common/complex diseases? The challenges in moving from the Mendelian situation to multi-factorial diseases or other human traits are many. First and foremost among these is genetic heterogeneity, in which mutations in many such relatively rare, but high-risk, genes give rise to nearly identical phenotypes. For example, in breast cancer, although *BRCA1* and *BRCA2* account for many of the striking multiple-case breast cancer families, recently it has become apparent through candidate gene resequencing studies that very rare mutations in at least five additional genes contribute to the unexplained familial aggregation [6]. Nevertheless, it is clear that the genetic basis of the majority of high-risk breast cancer pedigrees remains unresolved. A more extreme example is delineated by McClellan and King [7] for hereditary deafness in which no fewer than 48 genes have been demonstrated to have mutations predisposing to this condition. In addition to multiple loci, it appears to be a general feature of most populations that there are a large number of different highly penetrant mutations in any given disease susceptibility gene; In *BRCA2* for example, at least 2000 distinct pathogenic mutations have been reported, along with ∼800 other sequence variants whose pathogenicity is still uncertain (Breast Cancer Information Core database [8]).

Another complication for studies of common complex diseases is that even when a gene is segregating in a family and is responsible for the majority of the cases, there are likely to be one or more affected individuals that do not share the predisposing mutation since the disease is common by definition. Although often these may be distinguished from ‘genetic cases” by such factors as age at onset, severity, disease subtypes, etc. these phenocopies still cause complications for the study of complex traits by MPS. There is also the possibility that some apparent high-risk families are the result of aggregation of many common moderate-risk loci segregating within the family, mimicking an autosomal dominant pattern of inheritance, and/or an unusual cluster of environmentally caused (non-familial/genetic) cases of the disease.

While a number of others have investigated strategies for the statistical analysis of rare variants from candidate gene or whole exome/genome resequencing studies [9]–[13], there has not been a similar consideration of family-based designs in which the detection of comparatively higher penetrance alleles are the objective.

In general the success of sequence-based approaches (whether whole exome or whole genome or other targeted strategy) for identifying susceptibility genes will depend on the genetic architecture of the underlying disease. Some have argued that most of the so-called ‘missing heritability’ is due to as yet undiscovered relatively common variation with very small effects, while others have argued that evolutionary forces are more likely to generate large numbers of very rare mutations in key sets of genes, each conferring relatively high risks of disease [7], In a recent editorial, Cirulli and Goldstein [14] argue that until whole genome sequencing costs are sufficiently low to allow case-control approaches to be sufficiently powered, “the primary engines of discovery” are likely to be sequencing of individuals in families having multiple affected individuals; and selecting individuals from extremes of a trait distribution. Further, they outline the utility of using shared variants in families followed by further co-segregation studies of candidate variants, as well as bioinformatics analyses to narrow down the list of potential disease-associated variants. They concluded that “The development of appropriate test statistics that combine these different lines of evidence is a current priority for the field”.

In this paper, we illustrate these concepts using a simulated two-stage whole-exome MPS experiment of a hypothetical common disease and derive some general guidelines and considerations for the design of such studies.

## Methods

### Assumptions

#### The assumed genome

We first assume that there are 25,000 genes in the genome, each of which may have rare genetic variation unrelated to the disease. In our simulations, we take an entirely agnostic view and assume that each of these genes may harbor disease predisposing variants as well as unrelated variants. That is, we will not select genes of interest for a second stage based on pathways or function (although from a practical point, we might in fact prioritize for the analysis based on these factors). Based on the empirical data derived from published whole-exome sequencing studies [15], we assume that there are 400 rare missense variants (rms) and 20 truncating/splice junction (TSJ) non-disease related variants identified per sequenced exome.

For the ‘true” disease risk variants, we assume that these will be detected with a sensitivity of 90% i.e., that 90% of the sequence variants that are present in the genomic/exomic DNA will be detected by the particular Exome Capture/MPS platform used. We recognize that different types of variants (e.g., frameshifts vs. single nucleotide substitutions) may in fact differ slightly in their probability of detection; however, this is dependent on many factors and is not likely to affect our overall results. Although some current exon-capture systems do not cover the whole-exome, newer and developing products are approaching full exome-coverage, and with the addition of reverse strand sequencing of the targeted capture of the exons missed by the available kits from Agilent and Nimblegen, complete coverage of the exome will soon be achieved. However, we have assumed a somewhat lower value for sensitivity to account for other types of genetic variation that may not be detectable by exome sequencing approach (e.g., deep intronic variants causing a splicing abnormality; or cis-acting regulatory elements). For simplicity we further assume that true pathogenic mutations in each of the relevant disease genes are comprised of 50% rare missense (rms) changes, and 50% substitutions occurring either at consensus splice sites (both intronic and exonic) or are nonsense mutations or small insertions/deletions leading to premature truncation (TSJ). This reflects the uncertainty of knowing in advance the mutational spectrum of an unknown set of genes; for a subset of simulated conditions, we have examined the effect of higher or lower proportions of TSJ mutations. We further assume that each variant detected has been assessed for sequence quality and has been tested against the relevant databases to ensure that it is rare (<0.005). Lastly we assume that for the rare missense variants, we can employ a filter based on bioinformatics sequence analysis of conservation across species, severity of amino acid substitution, predicted effects on splicing, etc. that will exclude two-thirds of unrelated variants but only 10% of true pathogenic missense variants. For example, we might require that missense variants affect residues that are conserved in mammals and also be non-conservative, which for the BRCA1 and BRCA2 genes results in approximately these figures. This filter will reduce the number of extraneous variants at the expense of missing some true variants.

### Underlying Genetic Architecture

Because the underlying genetic architecture is likely to have a big effect on the optimal strategies to identify disease loci, we considered the following situations. First, we assumed that combined, the total set of rare high risk variants in the genome acting on the disease accounted for in aggregate a total of a familial relative risk of 1.33. For many of the common cancers this represents about 1/3 of the overall familial risk and approximately half of the unexplained risk. For diseases with stronger familial components such as many of the autoimmune disorders, this will be a lower proportion of the total genetic variance, but could be about the same proportion of the unexplained heritability since in many of those diseases variation at the MHC accounts for a substantial fraction. In our main simulations we assumed that the frequency of the disease in individuals without a high-risk allele was 0.02 and that the increased risk conferred by such mutations was 5×, 7.5×, 10×, 15×, or 20× this baseline risk. In all cases we assumed the combined frequency of such alleles in any given gene was 0.0001. The descriptions of the models are shown in Table 1. Based on this model, and assuming that the loci act multiplicatively on risk, it is straightforward to calculate the number of such loci to achieve the desired overall familial risk of 1.33 as *N = log(1.33)/log(λ_Rk_) where λ_Rk_* is the locus-specific familial risk to first-degree relatives as calculated from the allele frequencies, and genotype relative risks (see e.g., Skol et al. [16]) as shown in Table 1.

**Table 1:** Genetic Models Examined.

Model	GRR	Penetrance	FRR	No. of loci for FRR_total_ = 1.33
I	20	0.4	1.036	8
II	15	0.3	1.02	15
III	10	0.2	1.008	35
IV	7.5	0.15	1.0036	68
V	5	0.10	1.0016	179

Susceptibility allele frequency = 0.0001, sporadic rate = 0.02. GRR: genotype relative risk; FRR: familial relative risk.

### Basic strategies to be compared

**Figure 1:**
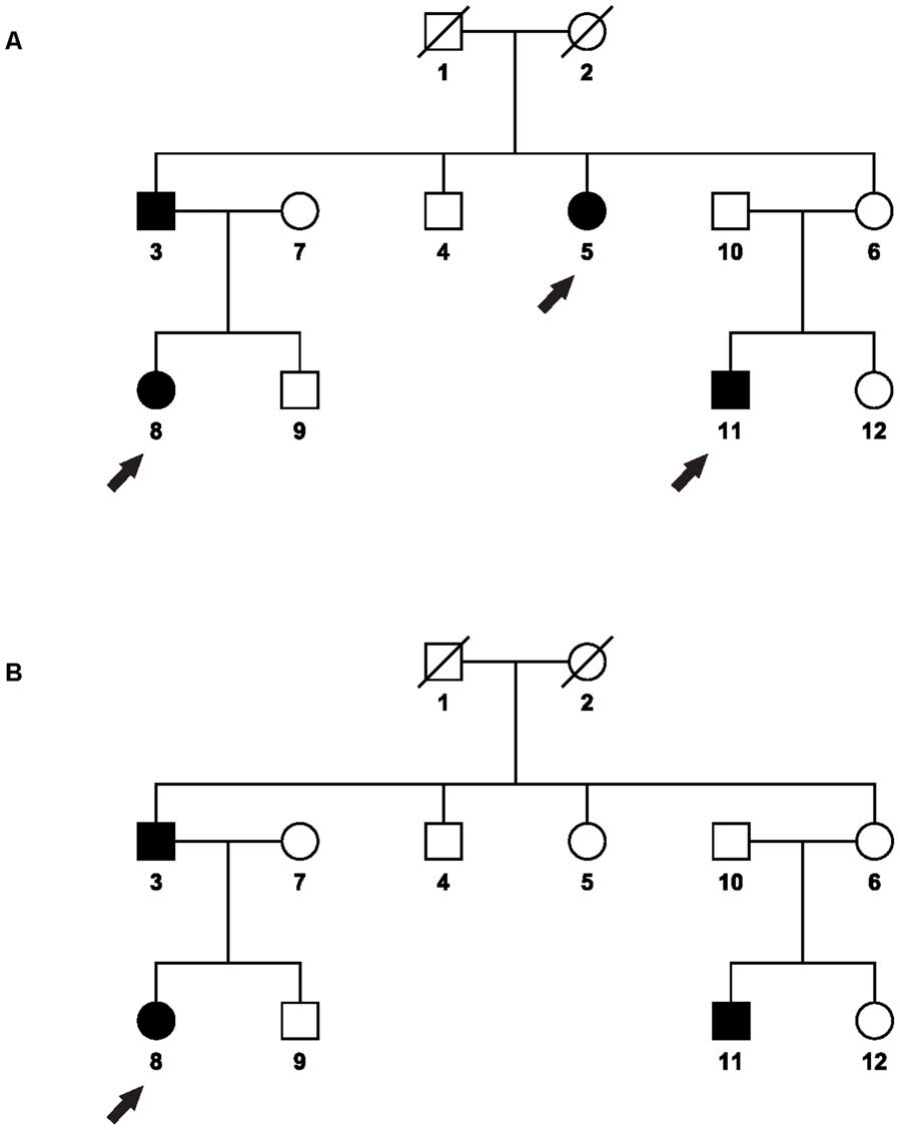
Pedigrees used in stage I and stage II. Panel A: pedigree structure for stage I. Individuals sequenced are indicated by an arrow: ID 8 is sequenced when one individual is sequenced; IDs 8 and 11 when two are sequenced; IDs 8, 5 and 11 when three are sequenced. Panel B: pedigree structure for stage II. ID 8 is analyzed.

Our basic design for all the simulations to be examined in this report is a two-stage design, where in the first stage, a relatively small number of very high-risk pedigrees (presumed to be likely to carry high-penetrance mutations in susceptibility gene) are analyzed by whole exome (or whole genome) MPS, followed by more conventional testing of candidate genes identified in stage I in a larger set of families that may have less dramatic family histories, by such techniques as High Resolution Melt analysis (HRM) or Denaturing High Performance Liquid Chromatography (DHPLC) which provide high-throughput mutation scanning at relatively low cost. Within this framework we compare designs in which one, two, or three individuals are sequenced per family. In all cases, we select those individuals who are most likely to be mutation carriers based on their phenotype and pedigree position. Figure 1a shows the pedigree structure assumed for the stage I analyses. Sequencing a single individual per family has lower up-front sequencing costs and typically it is easier to find appropriate families (we need only 1 sample with 3 ug of genomic DNA) in this approach; however, potentially more genes need to be screened in stage II. When multiple individuals are sequenced, there could be a loss of power if one of the two sequenced individuals is a phenocopy and only concordant variants are selected for further study; in addition, there are the increased costs of sequencing two or more individuals per family.

### Filters applied to variants/genes

In order to reduce the number of genes not associated with disease to be analyzed in the second stage, it will be necessary to apply one or more additional filtering steps in addition to the simple bioinformatics and frequency filters described previously. We can distinguish three classes of filter: concordance of the genetic variants and the disease in pedigrees; the requirement of observation of variants in the same gene in multiple families; and filters based on the type of variants observed. In addition, these three filtering strategies can be combined; we could for example only pass genes on to stage II that are observed to be concordant in two families, one of which is a truncating variant.

### Concordance Filter

For those situations in which we sample two or more individuals from each pedigree, the first filter will be to pass only variants and their respective genes for further validation if the variant is shared between all sequenced individuals. The efficiency of this filter depends on the degree of relationship between the individuals sequenced. In general, for two individuals related by degree D, we would exclude a proportion of (2^D+1^−2)/(2^D+1^−1) irrelevant variants. For cousins, for example, we would exclude 14/15 (93%) of such variants. When more than two individuals are sequenced, the situation is more complicated, but using the same approach similar probabilities can be calculated. In the case of the pedigree in Figure 1a, 45/46 (98%) of all non-disease related variants would be eliminated. Of course, it is also important that this process does not eliminate true susceptibility genes. The fraction of excluded true variants will depend on the genetic model, pedigree structure, and pedigree phenotypes. For example, under model II, the two cousins would be expected to be share a true disease susceptibility allele segregating in the family 73% of the time and all three sequenced individuals would be expected to share such an allele 67% of the time.

### Multiple Pedigree Filter

Beyond this “concordance filter” which, in any case, does not apply to the single individual sequence per pedigree approach, another strategy for distinguishing between the true susceptibility genes and genes containing spurious variants is the observation of variants in the same gene in multiple families. This filter can be tuned by requiring fewer or more observations in independent pedigrees. In our simulations, we have considered the situation where 1, 2, or 3 variants are required in a given gene for that gene to be passed to stage II. It should be noted that these filters apply only to variants that have passed the initial filters of sequence quality, frequency, and the bioinformatics screen as applied to rare missense variants in which 90% of true variants pass but only 30% of non-relevant variants are passed to the next step.

### Variant type filter

Although the bioinformatics filter reduces the number of missense/intronic variants that need to be considered, there were still be a large number of these variants that pass these initial filters. Given that truncating or consensus splice variants have a higher likelihood, *a priori*, of being pathogenic, it seems sensible that these could be given preference in choosing genes to sequence. The difficulty is that for some disease genes, the majority of pathogenic mutations are missense changes, which would result in potentially missing these genes.

Table 2 summarizes the filters that will be examined in this paper.

**Table 2:** Selected filters examined in the simulation.

Filter	One Case per pedigree	Two Cases per Pedigree
N1RV	All genes with a RV	All genes with a concordant RV
N1TS	All genes with a TSJ	All genes with a concordant TSJ
N2RV	All genes with RVs in 2+ pedigrees	All genes with RVs in 2+ pedigrees, at least one pedigrees concordant
N2TS	All genes with RVs in 2+ pedigrees, at least 1 RV is TSJ	All genes with RVs in 2+ pedigrees, at least 1 RV is TSJ, at least one pedigree concordant
N3RV	All genes with RVs in 3+ pedigrees	All genes with RVs in 3+ pedigrees, at least one pedigree concordant

RV: rare variant; TSJ: truncating/splice junction variant.

### Stage II Criteria

Each gene that passes through the series of filters outlined above will be sequenced in a set of families that are less highly selected than in stage I, but depending on the disease, would still have a reasonable probability of having a genetic basis. Figure 1b shows the pedigree used for the stage II simulations. We assume that we will use a low-cost, relatively high-throughput method such as High Resolution Melt analysis (HRM) or Denaturing High Performance Liquid Chromatography (DHPLC), or even potentially a second round of targeted capture MPS that sequences all the stage II genes at the same time and evaluates pools of samples from the stage II families. We take as our criteria for stage II confirmation of a given gene, that a qualifying rare missense or truncating/splice variant is found in e.g, 3 or more of the stage II families. Under this scenario, we can calculate the probability that the index case in each pedigree carries a mutation in a disease gene using e.g., the LINKAGE package [17], [18], and then using a simple binomial probability, calculate the probability that these would appear in 3 or more of 250 independent trials. The probabilities of the index case carrying a disease allele in one such gene are 0.061, 0.040, 0.019, 0.011, and 0.005 for models I–V respectively with corresponding probabilities of being validated with 3+ positive families are 1.0, 0.997, 0.86, 0.51, and 0.13, respectively. To calculate the probability that a gene that is unrelated to the disease of interest will meet the criteria of 3+ families having a qualifying variant in that gene we assume that there is a 0.1% chance that such a variant will be found in that gene given that we will apply the same frequency and bioinformatics filters in this stage as before. Note that this is still a 5-fold increase over the population allele frequency assumed for the disease-causing variants. For example, under this assumption the associated probability that a non-disease-related gene would have 3 or more qualifying variants in 250 pedigrees is 0.002. So even if 300 false genes are screened in stage II, the expected number of false positives is less than 1 and the probability of more than 2 false positives being validated in stage II is 0.023. Table 3 shows the probabilities of being validated for both true susceptibility genes and non-disease related genes for a range of sample sizes and thresholds for the number of observed variants.

**Table 3:** Effect of different sample sizes and validation thresholds on type I and type II error in stage II.

		Number of variants needed for validation		
Model	nPeds	≥2	≥3	≥4
Not Associated	150	0.010	0.0004	0.00002
	250	0.026	0.002	0.0001
	350	0.049	0.005	0.0005
I	150	1.0	0.99	0.98
	250	1.0	1.0	1.0
	350	1.0	1.0	1.0
II	150	0.98	0.94	0.85
	250	1.0	1.0	0.99
	350	1.0	1.0	1.0
III	150	0.78	0.54	0.32
	250	0.95	0.86	0.70
	350	0.99	0.96	0.90
IV	150	0.49	0.23	0.09
	250	0.76	0.52	0.30
	350	0.90	0.74	0.54
V	150	0.17	0.04	0.01
	250	0.36	0.13	0.04
	350	0.52	0.26	0.10

nPeds: number of pedigrees in stage II. Entries are the probability of meeting the specified validation criteria for a given sample size and model. Models are described in Table 1.

### Evaluation of Strategies and Filters

In choosing a strategy/filter combination, our goal is to maximize the number of “true” genes identified, at a minimum of cost/labor expended. A big part of this cost will be the unnecessary screening of false genes in a large number of individuals in stage II. The cost function we used to evaluate the strategies is as follows: We assume a cost of $4000 for each sequenced exome and a cost of $10 for mutation screening an average sized gene in each individual sample in stage II. For these calculations we assumed the middle sample size from table 3 of 250 pedigrees in stage II. The overall cost of the study is then:
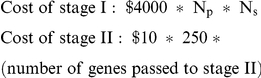
where N_p_ is the number of pedigrees in stage I, and N_s_ is the number of individuals sequenced per pedigree in stage I. The total cost is then the sum of the stage I and stage II costs. The essential feature that we want to capture here is the trade-off between the high cost of screening a single individual for the entire exome (or genome) compared to the cost of screening individually many promising candidate genes arising from the whole exome approach.

### Simulation Procedure

In order to assess the performance of the various strategies and filters under the assumed genetic models, we performed a simulation study as described below. The first step was to calculate the probabilities that each person in the pedigree carried a true risk allele under the given susceptibility model. We did this by using the program SLINK [19], [20] with 10,000 simulated pedigrees, using parameters corresponding to each genetic model, an assumed allele frequency of 0.0001 and complete linkage disequilibrium and no recombination between the disease allele and the presumed mutation (i.e., the disease allele is the variant being simulated). Using these probabilities we then select (though generation of a series of uniform-[0,1) random numbers) which of the N true genes (if any) were segregating in each family. Next, in a similar fashion the probability that the other individual sequenced in the pedigree is also carrying the mutation is used to determine if the pedigree is concordant for the chosen disease-associated variant. Then we assign 400 rare missense and 20 truncating/splice non-disease related variants randomly selected from 25,000 genes to each sequenced individual, keeping track of which of the 25,000 genes each variant is in, and where required, if it is concordant in the pedigree. This is repeated for each pedigree in the data set. Lastly we apply the designated additional filters to determine under each scenario which genes are passed to stage II. The previous steps were independently repeated 100 times and the number of true and false genes passed to stage II recorded for each replicate. These are then used to calculate the total cost of the study.

## Results

In the presentation of the results in the manuscript, we will focus on the key aspects of the analysis and findings and only present results for a selected set of filters and sample sizes (the detailed results for all models, filters, and sample sizes can be found in Table S1). Although in the tables to follow, we present only averages across the 100 replicates, the standard deviations as calculated from the 100 replicates were generally between 1 and 2 for the number of true genes identified and (as one would expect from this type of counting process) roughly the square root of the average number of false genes. For example for 30 pedigrees, and the filter requiring two variants in the same gene, under model II we identify on average 8.5 genes of the 15 true genes and 210 false genes with respective standard deviations of 1.3 and 13.5.

It is instructive to look at the number of “false” genes that are selected for screening in stage II, as this will in most cases have the greatest impact on the overall cost of the study. Table 4 provides these figures as a function of sample size for the five filters shown in table 2 under the scenarios of sequencing one, two, or three individuals per pedigree, and requiring at least one variant to be concordant when multiple individuals were sequenced. Because these false positives are independent of the disease model, they only have random variation across the various models and thus the figures provided are averages across the five models examined.

**Table 4:** Number of false genes passed to stage II for various filtering strategies, averaged across the five disease models.

Filter	N_s_	N_p_ = 10	N_p_ = 20	N_p_ = 30	N_p_ = 40	N_p_ = 60
N1RV	1	1359	2641	3857	5006	7119
	2	174	346	520	687	1022
	3	87	175	262	347	518
N1TS	1	199	396	591	785	1169
	2	25	50	75	100	149
	3	12	24	38	50	74
N2RV	1	34	138	305	527	1111
	2	15	60	133	225	460
	3	12	45	96	159	316
N2TS	1	9	37	83	144	309
	2	4	17	37	65	138
	3	3	13	28	48	102
N3RV	1	0	5	16	36	117
	2	1	5	18	41	123
	3	1	6	20	43	124

N_p_: number of pedigrees in stage I; N_s_: number of individuals sequenced per pedigree in stage I; Filters are described in Table 2.

The first line of table 4 points out the clear need to employ some sort of filtering strategy to reduce the number of potential genes that would need to be analyzed in stage II; even if we sequence two individuals and use concordance as a filter, for large numbers of pedigrees, the number of genes is quite large. In all cases, it is important to remember that these figures only include variants that have already passed through frequency filters (e.g., are not found in the 1000 Genomes Project database [21]) and bioinformatics filter that we assumed would eliminate 70% of non-disease related variants while passing 90% of true disease genes on to validation in stage II.

Table 5 examines the effects of sample size, number of individuals sequenced per pedigree, and filter on the number of true genes found and total study cost for Model II, in which there are 15 unknown disease genes in the genome, each conferring a 15-fold increased risk of disease.

**Table 5:** Number of true genes found and study cost as a function of filter, number of pedigrees, and number of sequenced individuals for model II (GRR = 15, number of susceptibility genes = 15).

nPeds	nCs	nExomes	N1RV		N1TS		N2RV		N2TS		N3RV	
			cost	nTrue	cost	nTrue	cost	nTrue	cost	nTrue	Cost	nTrue
10	1	10	3.5	6.98	0.5	4.11	0.1	1.80	0.1	1.24	0.0	0.31
10	2	20	0.5	5.32	0.1	3.06	0.1	1.73	0.1	1.34	0.1	0.31
10	3	30	0.4	5.10	0.2	2.71	0.2	1.93	0.1	1.33	0.1	0.32
20	1	20	6.7	10.87	1.1	7.07	0.4	5.40	0.2	4.12	0.1	1.85
20	2	40	1.0	8.72	0.3	5.24	0.3	5.15	0.2	4.25	0.2	2.21
20	3	60	0.7	8.17	0.3	4.93	0.4	5.59	0.3	4.10	0.3	2.39
30	1	30	9.8	12.97	1.6	9.09	0.9	8.34	0.3	6.58	0.2	4.15
30	2	60	1.6	10.95	0.4	7.54	0.6	8.41	0.3	6.95	0.3	4.53
30	3	90	1.0	10.45	0.5	6.92	0.6	8.34	0.4	6.78	0.4	4.93
40	1	40	12.7	13.86	2.2	10.67	1.5	10.76	0.5	9.26	0.3	6.67
40	2	80	2.1	12.33	0.6	8.64	0.9	10.64	0.5	9.21	0.4	7.37
40	3	120	1.4	11.65	0.6	8.41	0.9	10.46	0.6	9.26	0.6	7.70
60	1	60	18.1	14.69	3.2	12.61	3.0	13.26	1.0	12.03	0.6	10.92
60	2	120	3.1	13.93	0.9	11.22	1.7	13.33	0.9	11.69	0.8	11.17
80	1	80	22.9	14.90	4.2	13.66	5.0	14.53	1.7	13.39	1.0	13.24

GRR: genotype relative risk; nPeds: number of pedigrees sequenced; nCs: number of cases sequenced per pedigree; Cost is in million USD; nTrue: number of true susceptibility genes passed to the validation in stage II. Filters are described in Table 2.

Table 6 examines the study cost and the proportion of genes identified in stage I for a fixed sample size of 60 exomes sequenced (with differing numbers of sequenced individuals) for the five filters and five models of disease. These results demonstrate that the requirement that two or more pedigrees have variants in a given gene can effectively reduce the cost, with or without the concordance filter, and that the power lost due to this requirement depends on the expected number of true genes and the sample size. The higher the ratio of sample size to the expected number of genes, the smaller the decrease in power. From the table we can also see that requiring at least one variant be TSJ is another effective filter, under the assumption that half of the true disease variants are of this type. Note also that for models with many disease loci but where each has a smaller effect on disease risk, fewer genes are identified using any of the designs with cost <$1M, especially when considering the number of genes that are contributing. For example in the model IV where there are 68 susceptibility genes each conferring a relative risk of disease of 7.5-fold, the best designs only identify slightly over 10% of these 68 loci.

**Table 6:** Study cost and proportion of true genes passed to validation in stage II as a function of filter and genetic model, for selected sample sizes.

Model	nPeds	nCs	N1RV		N1TS		N2RV		N2TS		N3RV	
			cost	pr	cost	pr	cost	pr	cost	pr	cost	pr
GRR = 20,nGenes = 8	60	1	18.1	1.00	3.2	0.97	3.0	0.99	1.0	0.98	0.6	0.97
	30	2	1.6	0.91	0.4	0.74	0.6	0.86	0.3	0.76	0.3	0.66
	20	3	0.7	0.81	0.3	0.55	0.4	0.67	0.3	0.55	0.3	0.41
GRR = 15,nGenes = 15	60	1	18.1	0.98	3.2	0.84	3.0	0.88	1.0	0.80	0.6	0.73
	30	2	1.6	0.73	0.4	0.50	0.6	0.56	0.3	0.46	0.3	0.30
	20	3	0.7	0.54	0.3	0.33	0.4	0.37	0.3	0.27	0.3	0.16
GRR = 10,nGenes = 35	60	1	18.1	0.84	3.2	0.55	3.1	0.52	1.1	0.42	0.6	0.25
	30	2	1.6	0.39	0.4	0.21	0.6	0.20	0.3	0.17	0.3	0.07
	20	3	0.7	0.24	0.3	0.13	0.4	0.11	0.3	0.08	0.3	0.03
GRR = 7.5,nGenes = 68	60	1	18.1	0.65	3.2	0.35	3.1	0.28	1.0	0.20	0.5	0.09
	30	2	1.6	0.21	0.4	0.11	0.6	0.09	0.3	0.06	0.3	0.02
	20	3	0.7	0.12	0.3	0.06	0.4	0.05	0.3	0.03	0.3	0.01
GRR = 5,nGenes = 179	60	1	18.2	0.45	3.2	0.17	3.1	0.12	1.0	0.07	0.5	0.02
	30	2	1.6	0.08	0.4	0.04	0.6	0.03	0.3	0.02	0.3	0.00
	20	3	0.7	0.04	0.3	0.02	0.4	0.01	0.3	0.01	0.3	0.00

GRR: genotype relative risk; nGenes: number of susceptibility genes; nPeds: number of pedigrees sequenced; nCs: number of cases sequenced per pedigree; pr: proportion of true susceptibility genes passed to the validation in stage II; cost is in million USD. Filters are described in Table 2.

Only considering truncating variants may be a good strategy depending on the proportion of pathogenic variants that are of this type. In examining the results presented thus far, it is important to remember that we assumed that 50% of all disease alleles were assume to be those that result in a truncated (or null) protein or could be assumed to severely influence normal splicing of the gene. Because this may certainly vary between different disease genetic architectures, we have examined the effect of varying this proportion for select models. Here we will only consider two filters, and focus on the intermediate model with 15 genes each with rare alleles conferring a risk of 15× over population rates. In these cases we examine the effect of varying the proportion of pathogenic mutations in each gene that are of the TSJ variety from the 0.5 that we have assumed thus far, and look at values of 0.3 and 0.7 to determine the effect of this proportion on the efficiency of the various strategies. Table 7 shows the effect of these different assumptions on the number of true disease genes identified in stage I.

**Table 7:** Number of true genes identified as a function of proportion of pathogenic mutations that are type TSJ.

	N_p_(N_s_) = 20(1)		N_p_(N_s_) = 20(2)		N_p_(N_s_) = 40(1)	
	nTrue	Cost	nTrue	Cost	nTrue	Cost
Filter = N2RV pTSJ = 0.5	5.2	4.4	5.2	3.2	10.6	15.0
Filter = N1TS pTSJ = 0.3	4.9	a	3.3	a	7.9	a
Filter = N1TS pTSJ = 0.5	7.1	10.9	5.2	3.0	10.7	21.5
Filter = N1TS pTSJ = 0.7	8.7	a	6.7	a	12.5	a

pTSJ: proportion of pathogenic mutations that are type TSJ; N_p_: number of pedigrees in stage I; N_s_: number of individuals sequenced per pedigree in stage I. nTrue: number of true susceptibility genes passed to the validation in stage II; cost is in 100k USD.

a Cost does not vary with pTSJ, therefore the number should be equal to that for Filter = N1TS pTSJ = 0.5. Filters are described in Table 2.

## Discussion

In this set of simulations we have attempted to show some of the more important aspects and principles of design of studies involving MPS of common human disease. Our overall approach is modeled on the two-stage GWAS design in which cases and controls are assayed on a dense (500K→1M) SNP chips, followed by a second stage in which a smaller number of SNPs consisting of the top *N* (e.g., 10,000) SNPs (ranked by *p*-value) are tested on a (usually) larger independent set of cases and controls. For MPS studies our overall design is a first stage in which whole-exome sequencing is performed on samples from individuals in exceptionally high-risk families (or other extreme phenotype). In our case, the second stage is not a set of SNPs or variants, but a screen by sequencing of a relatively small number of genes that meet the criteria applied in the first stage, and in a larger number of individuals using a relatively inexpensive high-throughput screening strategy using methods such as HRM, DHPLC, or even targeted capture MPS. Within this framework we have attempted to look at the trade-off between the power to detect true disease loci against the vast number of false positives that will be generated in any MPS experiment. More importantly, we have examined the efficacy/efficiency of a variety of filtering strategies in reducing the number of false positives without dramatically affecting the power to detect the true disease susceptibility loci. Liu and Leal have also explored the strategies for two-stage designs in the context of whole-exome sequencing of a series of cases and controls with individual interesting variants or genes evaluated in a second stage [22]. In their case, the two primary strategies compared were to evaluate individual variants in the second stage or to evaluate through re-sequencing the genes in which those variants were identified. They concluded that sequence-based replication is generally advantageous if the stage I sample size is relatively small, as many variants will not be identified in the initial sequencing. The methods proposed by ourselves [13] and Price et al [10] both incorporate filters based on allele frequency and *in silico* analysis of sequence variants in order to create a more powerful aggregate test of the hypothesis that rare variants in a gene are contributing to disease risk than methods based on simply counting the numbers of variants observed in cases and controls as in e.g., [11]. The problem we address here differs from the case-control design examined in the above studies in at least two aspects. First, our assumption is that the individual disease alleles are much rarer than those proposed above and would not be amenable (even in aggregate) to a case-control approach; and second, we are considering the whole exome rather than a specific gene or pathway as is typically done in the case-control situation as analyzed above. It is not clear how these methods would work when applied to 20,000 genes; it is likely that the required sample size to detect the (even large) effects of many such alleles with correction for the multiple comparisons inherent in such a genome-wide approach would be prohibitive. Kryukov et al. [23] have specifically examined the power of whole-exome sequencing studies using extreme phenotypes and concluded that for reasonable affect sizes, detecting the effects of rare alleles in individual genes would be possible, although the sample sizes would be in the 1000s for whole-exome sequencing and the number of individuals that would need to be phenotyped to provide adequate selection of extremes would be substantially larger. Thus we focused our paper on the problem of vanishingly rare variants of large effect and the use of family studies to identify the specific genes harboring such variants.

Given that there are a large number of possible genetic architectures underlying each disease, and in most of these cases we can only make educated guesses about the true genetic basis. However, in the analyses presented here we have explicitly or implicitly assumed several key features. First, we assume that a substantial proportion of the “missing” genetic variance is due to individually rare alleles that confer moderate to high increased risk of disease (5–20 fold). Second, we have assumed that the pedigrees available for whole exome sequence analysis will be likely to be segregating a pathogenic mutation in one such gene, although not all cases in the pedigree are necessarily due to this mutation (i.e., phenocopies). Additionally we have assumed that each sequenced exome will contain a large number of rare missense variants that are independent of disease and a smaller number of protein truncating variants, even after filtering by frequency and by *in silico* analyses. We have assumed that this filter would reduce the number of rare missense variants by 70%; although this may seem somewhat arbitrary we note that our experience in analyzing a number of genes, shows that this level of filtering is easily achievable even using available multiple species protein sequence alignments. Further such a filter can be easily adjusted to provide more (or less) stringent filtering by requiring different degrees of evolutionary sequence conservation and/or more radical changes of the affected residue.

Taken as a whole, the results presented in Tables 4, 5, 6, and 7 demonstrate that while the choice of an appropriate strategy will depend on a variety of factors, the optimal degree of filtering will depend on the sample size as well as the choice of the number of individuals sequenced in each pedigree; there is no single optimal strategy. As Table 4 shows, when stringent filtering based on multiple variants in the same gene in different pedigrees is applied (e.g., N3RV) the number of false positives is approximately the same for a fixed number of pedigrees no matter how many individuals are sequenced, indicating that the concordance aspect is not as important since a given variant has to only be concordant in a single pedigree and so there is a balance between the number of exomes sequenced and the additional filtering, whereas for the looser filter N1RV there are large differences in the number of false genes as a function of the number of sequenced per pedigree. Table 4 also demonstrates that requiring multiple rare variants that are potentially pathogenic (based on a simple bioinformatics filter) in the same gene as a filter for selection to stage II sequencing is a very effective strategy in reducing the number of false positives (and hence the cost). Of course, this will also reduce the number of true genes identified, with the magnitude depending on the true underlying genetic architecture.

Under the models examined, one point that is evident from our results is that as the number of pedigrees increases, the cost of doing the study increases more rapidly than the increase in the number of susceptibility genes identified, particularly when only a single individual is sequenced per pedigree. This is true under a variety of different filtering strategies.

Often the choice of strategy will be determined by the availability of a sufficient number of suitable pedigrees and, beyond that by the ability to get sufficient quantity and quality of DNA from the appropriate members of the pedigree in the two or three individuals per pedigree case. In many cases it is easier to get larger number of pedigrees suitable for analysis in stage II. In this regard, it is useful that there are designs that are more or less equivalent in terms of cost and number of true genes identified using each strategy. In choosing which cases within a pedigree to sequence in stage I, there is typically a trade-off between power and false positive rate. If they are too closely related (e.g. siblings), the concordance filter cannot effectively exclude false positive genes; on the other hand, if they are too distantly related, particularly without intervening affected relatives and a common disease, the probability that the two individuals are not sharing a true high-risk mutation is increased, and the concordance filter will have a higher likelihood of rejecting a true disease gene.

We have made both explicit and implicit assumptions in our simulations, including the numbers of genes in the genome, the distribution of variants across those genes, the proportion of pathogenic variants of a given type, and the sensitivity of MPS sequencing in detecting true pathogenic variants. Although inaccuracies in these assumptions may affect some of the finer details of our results, we believe that the overall conclusions of our study are sound. Clearly the biggest factor influencing our ability to identify novel susceptibility alleles for complex human disease is the underlying genetic architecture which unfortunately is essentially unknowable, although often epidemiological and other data such as linkage studies, can provide some rough guides. If much of the genetic variance is due to many rare alleles of relatively modest effect in many different genes, and if the disease is common, it is likely that different approaches will have to be developed to identify these genes.

Our study shows that the choice of the appropriate design and filtering strategies will likely depend on many factors, and there is no “one-size fits all” recommendation. The choice of design depends on funds available, the ability of identifying high-risk families such as that typified in our simulations, as well as the ability to obtain DNA samples from the best individuals within each pedigree. Sequencing three cases per family does not add much additional variant filtering compared with the two individuals per pedigree case, and thus does not meaningfully reduce the overall cost. However, this strategy does result in lower power as a result of the exclusion of true genes due to the higher probability of one of the three cases being a phenocopy. Nevertheless, we note that there may be situations where it would be desirable to sequence three cases if, for example, only siblings were available. Nevertheless, our results provide some general guidelines that indicate that a reasonable fraction of moderate to high penetrance genes can be identified for complex diseases with practical and economical study designs. As costs of MPS sequencing drop for both whole exome and whole genome approaches, different strategies may become economically feasible. In particular, it may be possible to perform the second stage on larger numbers of genes using targeted sequence capture, or all available families could be screened in the first stage. In either case, there will still be a need for effective filtering strategies, particularly in the case of whole genome sequencing. Although the strategy employed and the sequencing method employed are clearly important, the key to success in identifying novel susceptibility for common disease will ultimately rely on the availability of large series of well-characterized families with many cases of the disease of interest and with the appropriate collections of biospecimens available for study. We recommend that before embarking on whole-exome or whole-genome studies in complex human diseases, careful consideration be given to the concepts discussed in this paper under a set of disease-specific plausible genetic models. We encourage interested readers to use the data in the supplemental data to repeat these analyses using relative costs of exome sequencing to candidate gene screening and sample sizes that are pertinent to their situation. To assist in this effort, the simulation program used in this study is available from the authors on request.

## Supporting Information

Table S1 Simulation results for all disease models, filters and sample sizes.
(XLS)
